# EU Joint Clinical Assessment: A Framework for Optimising Use with Cost-Effectiveness Decision-Making

**DOI:** 10.3390/jmahp13040052

**Published:** 2025-10-09

**Authors:** Adam Johns, André Andrade, Sukhvinder Johal, James Ryan

**Affiliations:** 1 HTA and Modelling Science, Oncology Business Unit, AstraZeneca, 08002 Barcelona, Spain; andre.andrade@astrazeneca.com; 2 HTA and Modelling Science, Oncology Business Unit, AstraZeneca, Cambridge CB2 1RY, UK; sukhvinder.johal@astrazeneca.com (S.J.); james.ryan@astrazeneca.com (J.R.)

**Keywords:** joint clinical assessment, cost effectiveness analysis, health technology assessment, comparative effectiveness, decision-making

## Abstract

The introduction of the European Union (EU) Joint Clinical Assessment (JCA) under Regulation (EU) 2021/2282 marks a transformative step in harmonizing health technology assessments (HTAs) across EU member states. This article explores the implications of JCA, particularly in oncology, for member states who utilize cost-effectiveness (CE) analysis and health technology developers (HTDs) who produce this evidence. The JCA framework attempts to standardise the assessment of relative clinical effectiveness and safety across the EU to input into national appraisals. Importantly, it excludes economic evaluations that may be required nationally, necessitating HTDs to align their CE models with the JCA PICO (Population/Intervention/Comparator/Outcome) parameters outlined by member states. This article discusses the challenges and opportunities for aligning JCA and CE modelling outcomes, contrasting evidence requirements between JCA and CE frameworks. It highlights the potential increase in complexity due to the diverse comparators in PICO surveys, necessitating the use of indirect comparison methodologies. It further underscores the importance of early communication between HTDs and HTA bodies to ensure timely, relevant, and pragmatic decision-making. By sharing national PICOs upfront to support national evidence generation, the JCA framework’s potential to aid high-quality decision-making and improve patient access to innovative medicines can be maximised.

## 1. Introduction

From January 2025, the European Union’s (EU) new regulation on health technology assessment (HTA), Regulation (EU) 2021/2282, established a common framework for joint clinical assessments (JCA). Under this regulation, the Joint Clinical Assessment (JCA) subgroup within the Member State Coordination Group on Health Technology Assessment is responsible for conducting centralised, EU-level assessments of the clinical effectiveness of new health technologies, primarily medicinal products and advanced therapy medicinal products, but also certain medical devices. JCA should provide a harmonised, EU-wide scientific analysis of the evidence on the relative clinical and safety effects of a health technology and the associated uncertainties. The JCA runs in parallel with the European Medicines Agency’s (EMA) regulatory evaluation process, although they are independent processes with different remits. The JCA submission dossier and report are intended to be given due consideration by member states in their national HTA processes [1].

The political ambition of the JCA framework is to improve access to medicines by harmonizing transparent HTA criteria at the EU level, standardizing the methodology used to assess the clinical benefit of a health technology compared to its best available alternative [2]. This analysis can inform decisions on the allocation of health care resources, including pricing and reimbursement, and access to health technologies at a national level [3]. While its stated goal is to reduce duplication and inform national HTA procedures, the JCA itself is focused on evaluating the relative clinical effectiveness and safety of new technologies relative to existing standards of care. The JCA cannot provide value judgements on the clinical benefit or relative safety, or rank endpoints themselves. These aspects remain the purview of each member state.

The JCA is therefore most aligned with the current clinical assessment undertaken by the Institut für Qualität und Wirtschaftlichkeit im Gesundheitswesen (IQWiG) in Germany, where, similarly to other markets where a formal clinical assessment is undertaken, its use could potentially replace their clinical assessment procedures. However, HTA systems within the EU vary, with most HTA bodies, including HTA collaborations such as the BeNeLuxA Initiative and Joint Nordic HTA Bodies, adopting a cost-effectiveness approach, typically focusing on quality adjusted life years (QALYs) as the measure of health benefit. Although clinical data is often regarded as transferable between countries, as seen with the use of global clinical trials and the EMA decision applying across the EU, this is not the case for economic analyses. For example, economic analysis will contain country specific data including resource use, unit cost, medical practice and management, country specific health state valuation sets, and national epidemiology, as well as different perspectives (health care payer versus societal). For these reasons of variations, the EU HTAR excludes economic analyses from the JCA component.

Therefore, health technology developers (HTDs) with an eligible technology will be required to not only provide a single JCA submission but also multiple national HTA submissions, including cost-effectiveness analysis for those countries requiring them. Although clinical effectiveness is an important component of cost-effectiveness based HTA systems, the JCA submission and report will be significantly larger than these national systems are used to, and it remains to be seen how and to what extent the JCA will be utilised.

It is, therefore, imperative for stakeholders to determine how to successfully align economic models and the JCA in a way that can reduce decision-uncertainty by addressing member states’ key questions, avoiding delays in national submissions and processes, and ultimately achieving faster and broader access to novel technologies for EU patients.

Unfortunately, the HTD has a relatively limited role in the JCA at a European level, as defined by the Coordination Group guidance and the Implementing Regulation, other than to notify of an EMA application and submit the JCA dossier [4,5]. However, they have a more active role in national HTA processes and appraisals. Therefore, as the first JCAs commence, this paper offers a practical framework to help both national HTA bodies and HTDs understand similarities and differences between JCA and cost-effectiveness assessments and align the JCA submission and report with economic analyses conducted at the national level. It focusses on six domains, informed by the Coordination Group’s JCA process, PICO and comparative assessment guidance documents, and considerations about how JCA may shape economic modelling. These domains are HTA process considerations, populations, comparators, outcomes (clinical, patient-reported, safety, and surrogacy considerations), comparative effectiveness, and, finally, innovations in modelling. Table 1 provides a summary of these key similarities, differences, and considerations for HTDs and HTA bodies from the JCA and CE perspectives.

## 2. Domains

### 2.1. Process Considerations

The PICO (Population/Intervention/Comparator/Outcome) survey is the first critical step in the JCA process. Upon submission of an application to the European Medicines Agency (EMA) for a medicine, the JCA assessors develop an assessment scope proposal. All member states are invited to respond to this proposal via a PICO survey, which gives them the opportunity to request the PICOs relevant for their countries. Once consolidated, this scope outlines the population, subpopulations, and subgroups to be analyzed, as well as the comparators and outcomes for the HTD to submit. The JCA scope therefore becomes the cornerstone of the developers’ HTA preparation process at both EU and national levels, including the decision-problem to be addressed nationally and subsequent considerations for development and preparation of economic models for national reimbursement submissions.

Fundamentally, CEA is also traditionally based on a national PICO. For a JCA technology, this PICO will now be derived many months before marketing authorisation rather than around the time marketing authorisation is obtained and should become more explicit across each member state. However, economic model development and input parametrisation can be a lengthy process, and so to realise the benefit of overall earlier HTA process starts and quicker decision-making that the JCA offers, it will be important to submit this additional national economic, and where required, clinical evidence in a timely manner.

Early engagement between the national HTA body and the HTD before the PICO survey will bring mutual benefits in understanding the disease area, current clinical practice and downstream requirements for national decision-making, including cost-effectiveness model requirements. The member state can then make more informed input into the JCA scope, ensuring relevance to their national HTA. In addition, it is imperative that the member state communicates their national PICOs to the HTD once the final JCA scope is provided. Such early exchanges mean the HTD can ensure that relevant evidence can be produced and that the economic model developed is fit-for-purpose, reducing additional requests from local assessors. Without this information, the HTD may submit economic analyses based on the wrong PICOs, increasing business uncertainty for the HTD but, more importantly, adding additional work for local assessors and potential delays in the process, negating one of the benefits of the JCA. We also recognize that leading up to national assessments, new comparators may become available and routinely used in a country or the population may change due to the final marketing authorisation at the end of the EMA process. Furthermore, additional outcomes may be required, such as nonclinical outcomes or a different measure of an outcome compared to the JCA outcomes. In such cases, engagement at the earliest opportunity between the HTD and national HTA body will be required so such evidence can be developed and incorporated by the HTD.

Finally, it is important to distinguish between the JCA PICOs and the national PICOs. The additive process adopted by the Coordination Group as well as the combination of populations, subgroups, comparators, and outcomes can lead to a substantial number of PICOs at the EU level, and previous predictions have estimated a need to conduct thousands of separate analyses [6]. Whilst it may be feasible for an HTD to have a common economic model structure across countries, a single economic model that addresses all EU PICOs and incorporates all requested analyses at once would be impractical and inefficient and could increase decision-maker burden and uncertainty with excessive options and analyses that would not align with their specific clinical context and needs. Given that economic analyses are local, it is imperative that any national economic model addresses the specific PICOs requested by that national HTA body.

### 2.2. Populations

While economic considerations to evaluate the cost-effectiveness of PICOs most deserving of reimbursement are important to consider, it would be contrary to evidence-based medicine principles to use these to pre-determine what is to be analyzed to achieve cost-effectiveness. Based on insight from the Coordination Group’s PICO exercises, the overall JCA population is the population in the label, and subpopulations are patient groups within this labelled population [7]. The selection of populations and subpopulations in the JCA PICO scoping, typically referred to as patient subgroups in many HTA systems, should be driven by robust scientific and clinical considerations. As the JCA is based on clinical criteria, economic factors such as budget impact should not influence choice of population at the PICO stage.

For national decision-making, clinical practice and demographics vary across Europe, so populations included within the economic model should be specific to relevant clinical populations for that country identified in the JCA scope. At a national level, where the overall population is not deemed cost-effective, relevant subpopulations for decision-making on who to provide access for should be based on relevant treatment effect modifiers or prognostic factors rather than factors that cannot be demonstrated to influence treatment outcomes. The exception arises when populations may not be distinct through prognostic factors or treatment effect modifiers, but where they differ in terms of best available treatment alternative and/or subsequent treatments. In such cases, it may be appropriate to subdivide populations based on comparators.

### 2.3. Comparators

With diversity in health care systems and a dynamic treatment landscape in oncology, it is expected that each JCA will have multiple comparators, irrespective of therapy area. The national economic model should typically contain the main comparators for each population required by that country, which in turn should be based on the therapy or therapies most likely to be replaced by the new technology under the expected EMA label. Due to diversity in clinical practice, indirect comparisons will be imperative for both the JCA and usage at a national level. We discuss considerations for establishing comparative effectiveness below in relation to economic modelling.

### 2.4. Outcomes: Clinical

Clinical outcomes are important for both the JCA and cost-effectiveness in HTA countries. They form one important set of outcomes in the JCA to establish the relative effect of new technologies. In economic analyses, they are used in the first instance to establish clinical benefit over the comparator. However, in the economic model, they may help define health states and the time in each state to estimate quality-adjusted life years (QALYs). The outcomes included in the JCA dossier are ultimately chosen by the member states, which may include additional outcomes not captured in the pivotal trial(s). It is, therefore, important that the key clinical outcomes that may be required for member states’ cost-effectiveness assessment are requested at the JCA stage to be able to maximize the use of the JCA report.

For both JCA and CEA in the oncology context, the traditional core clinical outcomes of overall survival (OS) and progression/disease/relapse-free survival (PFS/DFS/RFS) will likely form the cornerstones of both assessments. In the context of JCA, these endpoint measures are likely based only on the observed data in the clinical trial. In contrast, for CE models, this data must be extrapolated beyond the observed trial data time horizon to evaluate the long-term clinical, health, and economic value of the technology. In addition, adjustments for potential sources of bias such as treatment switching or imbalances in the use of subsequent therapies may be needed for models, which may require national clinical pathway considerations [8]. While the JCA report should acknowledge appropriate methodology to reduce uncertainty where an outcome may be confounded and report that potential bias if it cannot be adjusted for, it is possible that the JCA dossier will not include extrapolations as these are often required to inform the longer time horizon of economic analyses.

### 2.5. Outcomes: Health Related Quality of Life (HRQoL) and Symptoms

It is anticipated that patient-reported outcomes (PROs), capturing how a patient feels and functions, will be a core component of the JCA dossier, measuring the impact of both HRQoL and symptoms. As directly indicative of the patient experience, it is critical to highlight these outcomes. As the Arzneimittelmarkt–Neuordnungsgesetz (AMNOG, German Pharmaceuticals Market Reorganisation Act) process has demonstrated, PROs are an important component of added benefit valuations of new medicines.

While PROs can support the relative clinical value of a technology, their use within economic modelling is limited. In some cases, they may define a particular health state, for example, in treatments that may reduce pain. However, in many economic models, especially in oncology where health states are often clinically defined, their direct use is limited. Furthermore, the cost-utility modelling framework used by many HTA systems is based on QALYs, which typically use health state utilities derived from the EuroQoL-5D questionnaire (EQ-5D) to offer a broadly generalizable assessment of a society’s preferences of a given health state. Utilities, unlike PROs, do not reflect the patient’s own valuation of their health state. Therefore, the Visual Analogue Scale (VAS) and changes in EQ-5D domain scores are relevant for the JCA, as they represent direct patient assessment, whereas the EQ-5D utility index should remain specific to national-level economic evaluations and not be requested at the JCA level.

PROs in the JCA provide an opportunity for national HTA bodies to consider the patient impact explicitly in their value frameworks. They should also play a crucial role in validating the health state utility values used in a model for both HTDs and HTA bodies. For example, an improvement in PROs for a new technology or a delay in deterioration should be considered in modelling assumptions around health state utility.

### 2.6. Outcomes: Safety

The JCA methods guide on outcomes asks for extensive safety analyses, not just in the overall population but across subpopulations [9]. This requirement most closely resembles German AMNOG requirements, although research has shown that the majority proportion of these analyses are not used or do not impact decisions [10]. By applying this across all PICOs, safety analyses can potentially lead to hundreds of pages of safety data across direct and indirect comparisons. If member states request the full inclusion of all JCA safety evidence within economic models, contrary to what is currently required by such HTA systems, there is potential to add significant additional complexity with very little benefit for decision-making. Therefore, member states should pursue a pragmatic approach to addressing JCA safety evidence.

Whilst safety is an important component from both a regulatory assessment of risk-benefit of a new medicine and for patient–clinician decision-making, it is less likely to influence cost-effectiveness model results, particularly in oncology. In general, HTA cost-effectiveness guidance typically involves incorporating the costs and health outcomes of adverse events (AEs), adjusted for likelihood of the event occurring. These costs are likely to be relatively low compared to the costs of the overall episode of care; in oncology, grade 3–4 AEs are typically the only AEs associated with a direct health system cost, and as these AEs generally resolve within a short period or result in treatment discontinuation, the cost is likely to be short-term [11]. While grade 1–2 AEs are undoubtedly patient-relevant and can contribute significantly to the experience of the patient, they are typically managed in a primary-care setting and are of comparatively low cost in the context of oncology treatment. The health status impact of these AEs, as well as those of grade 3–4, can be addressed in an economic model by including health state utility evidence representative of both treatment and state. Thus, while important in a clinical assessment, most safety evidence included in the JCA is unlikely to meaningfully influence the outcome of an economic model.

While a pragmatic and parsimonious approach to AEs is likely the best approach to aligning JCA and modelling needs, there are certain cases where additional AE evidence may be needed for an economic model. Firstly, the EMA may specifically call out adverse events of special interest during regulatory assessment; these may warrant direct consideration in the economic model. Additionally, where a country adopts a societal perspective in their national HTA framework, models should include consideration of the additional impact of adverse events on societal and carer costs and utilities.

### 2.7. Outcomes: Surrogacy Considerations

Where an endpoint is requested in the JCA but is not available at the time, for example, due to immaturity of that endpoint, the HTD can use surrogacy methodology to demonstrate how another endpoint (e.g., response rate or disease-free survival) effects the requested endpoint. An outcome of interest is not just restricted to overall survival but may also include other relevant endpoints of interest (e.g., cure, avoidance of surgery, improvement in health-related quality of life).

The JCA methodology on outcomes [9] emphasises the need for meta-analysis of several randomised control trials with a high correlation between the surrogate and the clinical endpoint of at least 0.85, reflecting Germany’s IQWiG stance on surrogacy [12]. However, for cost-effectiveness countries, the modelling framework itself can support prediction of longer-term outcomes. This modelling framework allows for both discussion on the relationship between endpoints and survival extrapolation techniques, contrasting with strict surrogacy threshold analysis which may never be achievable due to time to develop the evidence, confounding and new mechanisms of action. Furthermore, such surrogacy thresholds may create an ethical dilemma because they require evidence from other trials, which may be not available in sufficient quantity in rarer disease or harder to treat indications that have been poorly served.

The JCA methodology guide acknowledges that different HTA bodies may have different approaches and considerations. To address challenges with surrogacy analysis, and support consistency in approach across HTA bodies, the National Institute for Health and Care Excellence (NICE) in the UK, in collaboration with other international HTA bodies, including Zorginstituut Nederland, have recently developed a White Paper on surrogate endpoints for cost-effectiveness analysis in HTA which provides a set recommendations designed to be used alongside existing economic modelling guidance when using a surrogate endpoint, and includes considerations around definition, justification, adoption, statistical validation, incorporation, reporting, and approaches to quantify and present uncertainty [13]. We encourage HTDs and national HTA bodies to consider this White Paper when interpreting results from the JCA submission and report.

### 2.8. Comparative Effectiveness

One of the objectives of the JCA is to establish the relative effectiveness of a new technology compared to what is currently used in practice. Where evidence is available, this can be achieved through direct, head-to-head, analyses from a clinical trial. Where a comparator is requested that was not included in the trial, indirect comparison methods are an important tool to increase confidence in estimates and reduce uncertainty compared to undertaking naïve comparisons.

Whereas the JCA may focus on relative effect statistics such as the hazard ratio, additional factors need to be considered when assessing comparative effect within an economic framework, particularly in oncology. In cost-effectiveness HTA systems, the expected relative benefit is typically of more interest than the relative effect, as demonstrated by area under the extrapolated survival curve analysis in oncology models. Although the JCA methods guidance allows advanced indirect comparison techniques, the preference remains for methods that maintain randomisation such as the Bucher method [14]. However, for economic modelling, consideration should also be given to the assumptions being made in the JCA ITC methodology, and how they are applied within the economic model, particularly during periods of extrapolation, to ensure that these are statistically and clinically plausible.

As oncology landscapes become increasingly targeted and driven by innovative mechanisms of action it is likely that assumptions around the proportionality of the long-term hazard in a network meta-analysis (NMA) between existing and innovative therapies may not be met, as the introduction of immuno-oncology demonstrates [15]. This points toward the need for greater adoption of non-proportional methods such as piecewise NMA or fractional polynomials within both JCA and modelling frameworks. While adding complexity, these methods are nonetheless important to consider as long-term trends in the hazard function for survival is a key driver of CE model results. Under JCA, HTDs and HTA bodies will need to be comfortable with these methodologies and, while still striving for parsimony, should be willing to both develop and assess non-proportional NMAs as a matter of course. NMA methods to address non-proportional hazards may ultimately deliver more accurate and relevant evidence to member state HTA bodies. JCA assessors can help support this development by accepting methodologies which are more relevant in addressing PICOs and meeting the needs of member HTAs within a cost-effectiveness framework.

A second consideration when interpreting the JCA outputs is the potential generalisability of an endpoint, such as OS, due to treatment switching or subsequent treatments. For evidence to reflect national clinical practice, adjustments may need to be made to reduce this confounding. Such adjustments also need to be carried through in indirect comparisons, where appropriate, to ensure a more reliable prediction of incremental benefit in the national setting.

### 2.9. Innovation in Modelling

While parsimony in development of models remains an overriding principle for HTDs and HTA bodies, JCA and dynamic treatment landscapes have the potential to increase the complexity and volume of modelling, resulting in an increasing need for technical capability and resource for both HTDs and national HTA bodies.

Developing a “one size fits all” model incorporating all PICOs may appear a seemingly simpler option than developing specific models for each member HTA body; however a master JCA model incorporating all PICOs, evidence syntheses and complex methodologies such as non-proportional hazards NMA is likely to be computationally intensive and significantly harder for national HTA assessors to evaluate and quality check, as well distracting from their national PICOs for the purposes of decision-making. Furthermore, there will be local evidence considerations which influence the applicability of an economic model, such as the availability of subsequent treatments or adoption of a societal perspective; therefore, an economic model will almost always require local adaptation. Efficiency can be gained by adopting a consistent economic model structure for a specific new technology across countries. Furthermore, coding automation and support from emerging large language models (LLMs) may have the potential to assist with the task of adapting and tailoring models and contextualising reports in the future [16] to member state specific HTA PICOs in a timely manner. This may require use of modelling software beyond the capabilities of MS Excel.

Member states can help HTDs address the complexity of modelling under the JCA framework by keeping an open mind to technical innovation in model development and delivery, and by maintaining and strengthening the technical capabilities of their reviewers. Emerging areas of technical competency for reviewers could include evaluation of models in code-based platforms which allow for more automation in model development (such as R or Python), and use of cloud computing and parallel processing to support computationally intensive models. Although these technical demands will require new approaches, these methods can be developed via academic-HTD-HTA partnerships. Just as a consistent JCA framework promises to harmonize European HTA, research partnerships to advance the needed methodologies for modelling can ensure that the framework is able to meet the goal of more timely and equitable access for patient access. Although economic models are excluded from the JCA itself, the voluntary cooperation of the EU HTA Regulation itself, and programmes such as Support Utilisation of Sustainable and Tailored Innovative methods for HTA (SUSTAIN-HTA) [17] and the Innovative Medicines Initiative [18] could help support such future innovation.

## 3. Conclusions

The EU JCA framework represents a significant advance in aligning clinical evidence assessment across Europe, with the promise of stopping clinical assessment duplication across member states, streamlining HTA and associated decision-making, and improving sustainable and equitable patient access to innovative therapies. However, its successful integration with cost-effectiveness oriented HTA hinges on the HTDs’ ability to anticipate and align with the PICO requirements specified by member states. While the JCA focuses on clinical outcomes and statistical certainty, economic assessments continue to require robust models that incorporate relevant, extrapolated data to reflect health outcome and economic benefit over a longer time horizon whilst addressing structural, stochastic and parameter uncertainty.

Table 2 summarizes our recommendations to ensuring the JCA is maximised to its potential within cost-effectiveness HTA countries, whilst acknowledging that the remit of JCA as a scientific statistical assessment of clinical and safety outcomes differs to the decision-making remit of national HTA bodies. Importantly, close collaboration and communication between HTDs and national HTA bodies during both the JCA and national procedures will help ensure that the clinical and economic assessments synergistically address key national questions without adding unnecessary overwhelming complexity. Embracing methodological innovations and fostering flexible, pragmatic approaches will be pivotal in capitalizing on the JCA framework’s potential to enhance health outcomes while maintaining economic precision and timeliness.

## Figures and Tables

**Table 1:** Summary of considerations for HTDs and HTA bodies from an oncology perspective.

Domain	JCA Perspective	CEA Perspective	Considerations for HTDs	Considerations for HTA Bodies
**HTA process**	JCA scope contains all national PICOs. Scoping starts at EMA submission validation.	Economic analyses are country-specific and should reflect national PICO to ensure relevance. PICO will be produced earlier than normal.	Engage with the HTA body before JCA scoping to understand national needs and support maximising the relevance of the JCA to the national HTA body.	Share national PICO as soon as possible to provide sufficient time for the HTD to produce the most relevant economic evidence package supported by the JCA.
**Populations**	Additive approach across all Member States. As a JCA, they should focus on clinically relevant criteria rather than economic criteria.	Reflect specific national PICO populations requested at a JCA level and label.	Understand and statistically and clinically validate effect modifiers. Consider natural history of disease by population relevant to specific country.	If subpopulations required, request populations based on clinically verifiable prognostic factors/effect modifiers.
**Comparators**	Additive approach across all Member States.	Focus on national requested comparator (s) for (sub)population of interest.	Early engagement with national HTA bodies to inform, with evidence, local clinical practice and determine the most widely used comparator(s).	Consider what comparator would be replaced in practice if the new technology were to be reimbursed.
**Outcomes:** Clinical	A key component of the JCA, focussing on the observed clinical study duration and statistical certainty.	Contribute to both clinical benefit assessment as well as economic model structure. Need to be extrapolated (usually over a lifetime) and, where appropriate, adjusted for treatment switching and subsequent therapies depending on clinical and national context.	Assure alignment between JCA outcomes and model-based clinical data analyses and assumptions (extrapolations, adjustments for treatment switching).	Ensure PICO survey input includes the key endpoints needed for CE analyses. Where relevant, validate clinical assumptions in economic model align with JCA dossier/report.
**Outcomes:** Patient-Reported	HRQoL and symptom/morbidity-focussed. Analysis typically based on improvement rates or time to deterioration defined my minimally important difference. The EQ-5D VAS and change in domain scores are the most relevant measures for JCA as they are patient-reported.	Economic measure of health benefit estimated by QALY, composed of health state utility value and time in health states. The EQ-5D health state utility is the most applicable measure. In some cases, such as pain, PROs may define a health state in the economic model.	Ensure health state utility assumptions align with JCA evidence from PROs. Where no health state utility instrument is included in trial, consider mapping from PROs collected using a validated algorithm to estimate economic model utility values.	Consider role of PROs as a standalone measure of benefit in appraisal. Use JCA PRO evidence to validate or sense-check utility choices and assumptions made by the HTD.
**Outcomes:** Safety	Relative safety an important focus of the JCA. Guidance document request safety analyses at a “micro” level. not requested in many HTA markets.	Economic models do not model all the safety analyses requested by the JCA. In oncology, Grade 3–4 events are typically included but safety has a relatively limited impact on cost-effectiveness results. May be more impactful in some other disease areas or where treatments are comparable in effect.	Strive for simplicity in modelling safety while not overlooking key implications of JCA safety analyses, e.g., Grade 3 or 4, high frequency or costly to manage.	Consider if adding in extra complexity for completeness will fundamentally change the decision being made. Concentrate on the main drivers of the cost-effectiveness outcomes.
**Outcomes:** Surrogacy	Prefers high correlation between the surrogate and the clinical endpoint of at least 0.85. Acknowledges that surrogacy interpretation may differ between HTA bodies.	The modelling framework itself, along with survival extrapolation techniques in oncology, supports prediction of longer-term outcomes.	Be aware that surrogacy requirements may differ between member states and between clinical benefit and economic benefit HTA systems. Consider clinical validation to support assumptions.	Allow for inclusion of additional surrogacy evidence and consider NICE et al. surrogacy white paper.
**Comparative effectiveness:** Indirect Evidence	Comprehensive use of NMA and evidence synthesis to provide relevant comparisons for all PICOs. Preference for techniques maintaining randomisation, e.g., Bucher Based on observed period from data sets, with no consideration for long-term unobserved periods	Important to address the proportional hazard assumption (PHA) between comparators, particularly when modelling long term survival outcomes. Where the PHA does not hold, address methodologically with techniques such as piecewise HRs/fractional polynomials	Be comfortable with modelling (HTDs) and evaluating (HTA bodies) the results of non-proportional evidence synthesis. Accept that in some cases the national approach may differ from the JCA approach, particularly in oncology or other disease reliant on survival analysis. Consider need to account for bias in outcome results when undertaking or assessing indirect comparisons (e.g., to account for treatment switching or other confounders that need to be accounted for in the ITC).	

**Table 2:** Recommendations for utilising the JCA within cost-effectiveness HTA.

Recommendations
PICOs requested by member states for the JCA should be shared with the HTD at the earliest possible point to ensure timely, local evidence is provided, including economic models.
2. Populations should be selected based on clinical criteria and not economic criteria such as budget impact.
3. To ensure relevance of any economic assessment, comparators should be limited to approved therapies most likely to be replaced in clinical practice by the health technology, rather than all available therapies for a given population.
4. To maximise JCA usage, member states should request those eligible clinical outcomes for relative effectiveness estimates in the JCA scoping phase that are likely to be needed to inform economic model development.
5. HTDs and HTA bodies should be aware of the potential for biased results when the ITT analysis principle is applied; where confounding is present, appropriate adjustments should be considered and provided in national HTA, if not already done at the JCA level.
6. Patient-reported outcomes from the JCA should be used to validate modelling assumptions and inform health state utility values.
7. Within an economic model framework, adverse events (AEs) should typically be based on the overall population, utilizing the safety analysis set and focusing on the most important Grade 3–4 AEs.
8. Where appropriate, additional clinical analyses and methodologies may be necessary beyond those used for the JCA when undertaking economic modelling, for example, survival extrapolation and alternative indirect comparison techniques.
9. When interpreting intermediate or surrogate endpoints from the JCA report within an economic modelling framework, HTDs and HTA bodies should consider international collaborative HTA agencies recommendations on surrogate endpoints for CEA.

## Data Availability

No new data were created or analyzed in this study. Data sharing is not applicable to this article.

## Abbreviations and Acronyms

AEs	Adverse Events
AMNOG	Arzneimittelmarkt-Neuordnungsgesetz (German Pharmaceuticals Market Reorganisation Act)
CE	Cost Effectiveness
EMA	European Medicines Agency
EQ-5D	EuroQoL-5D questionnaire
EU	European Union
HTDs	Health Technology Developers
HRQoL	Health-Related Quality of Life
IQWiG	Institut für Qualität und Wirtschaftlichkeit im Gesundheitswesen (German Institute for Quality and Efficiency in Health Care)
LLMs	Large Language Models
JCA	Joint Clinical Assessment
NICE	National Institute for Health and Care Excellence
NMA	Network Meta-Analysis
PICO	Population/Intervention/Comparator/Outcome
QALYs	Quality Adjusted Life Years
OS	Overall Survival
PFS/DFS/RFS	Progression/Disease/Relapse-Free Survival
VAS	Visual Analogue Scale
